# *Bacillus licheniformis* SA03 Confers Increased Saline–Alkaline Tolerance in *Chrysanthemum* Plants by Induction of Abscisic Acid Accumulation

**DOI:** 10.3389/fpls.2017.01143

**Published:** 2017-06-29

**Authors:** Cheng Zhou, Lin Zhu, Yue Xie, Feiyue Li, Xin Xiao, Zhongyou Ma, Jianfei Wang

**Affiliations:** ^1^Key Laboratory of Bio-organic Fertilizer Creation, Ministry of Agriculture, Anhui Science and Technology UniversityBengbu, China; ^2^School of Life Science and Technology, Tongji UniversityShanghai, China

**Keywords:** soil alkalinity, rhizobacteria, low iron availability, iron acquisition, oxidative damages

## Abstract

Soil saline-alkalization is a major abiotic stress that leads to low iron (Fe) availability and high toxicity of sodium ions (Na^+^) for plants. It has recently been shown that plant growth promoting rhizobacteria (PGPR) can enhance the ability of plants to tolerate multiple abiotic stresses such as drought, salinity, and nutrient deficiency. However, the possible involvement of PGPR in improving saline–alkaline tolerance of plants and the underlying mechanisms remain largely unknown. In this study, we investigated the effects of *Bacillus licheniformis* (strain SA03) on the growth of *Chrysanthemum* plants under saline–alkaline conditions. Our results revealed that inoculation with SA03 alleviated saline–alkaline stress in plants with increased survival rates, photosynthesis and biomass. The inoculated plants accumulated more Fe and lower Na^+^ concentrations under saline–alkaline stress compared with the non-inoculated plants. RNA-Sequencing analyses further revealed that SA03 significantly activated abiotic stress- and Fe acquisition-related pathways in the stress-treated plants. However, SA03 failed to increase saline–alkaline tolerance in plants when cellular abscisic acid (ABA) and nitric oxide (NO) synthesis were inhibited by treatment with fluridone (FLU) and 2-(4-carboxyphenyl)-4,4,5,5-tetramethylimidazoline-1-oxyl-3-oxide (c-PTIO), respectively. Importantly, we also found that NO acted downstream of SA03-induced ABA to activate a series of adaptive responses in host plants under saline–alkaline stress. These findings demonstrated the potential roles of *B. licheniformis* SA03 in enhancing saline–alkaline tolerance of plants and highlighted the intricate integration of microbial signaling in regulating cellular Fe and Na^+^ accumulation.

## Introduction

Plants as sessile organisms cannot escape from negative effects imposed by detrimental environments such as high salinity, drought, freezing, high temperature, and flooding. Soil salinity has increasingly become one of major abiotic stresses that constraint plant growth worldwide (Frommer et al., 1999). In nature, soil salinity and alkalinity often occurs simultaneously due to the complexity of soils (Zhang et al., 2015). Approximately half of the saline soils in earth’s crust contain NaHCO_3_ and Na_2_CO_3_, which are the main factors that contribute to soil alkalinity (Yang et al., 2009). Excess Na^+^ and high pH value in saline–alkaline soils cause considerable damages to plant growth and development, whereas most plant species are more susceptible to high pH soils (more than 8.0) than saline soils (Tang et al., 2014; Li et al., 2016). High soil pH adversely affects seed germination, root cell structure and functions, the availability and absorption of nutrient elements, thereby leading to a remarkable decrease in crop yield and quality (Tang et al., 2014; Zhou et al., 2016a). According to the statistics, there is more than 800 million hectare of saline–alkaline soils in the world (Martinez-Beltran and Manzur, 2005). Hence, it is an urgent need to develop effective strategies to enhance the ability of plants to tolerate saline–alkaline conditions.

Saline–alkaline soils are generally characterized by high pH value and Na^+^ toxicity (Abadía et al., 2011; Tang et al., 2014; Li et al., 2016). High pH value decreases the solubility of soil iron (Fe), and most of Fe occurs in the insoluble form of Fe^3+^, which is not easily absorbed by plants (Romera and Alcántara, 2004). Upon exposure to alkaline stress, plants often exhibit typical symptoms of Fe deficiency-induced chlorosis (Gong et al., 2014; Zhou et al., 2016a). Emerging evidence has indicated that the enhanced Fe acquisition can confer greater tolerance of plants to alkaline stress (Li et al., 2016; Zhou et al., 2016a). During long-term evolution, different plant species develop strategy I and II mechanisms to adapt to Fe deficient conditions, respectively (Marschner and Römheld, 1994). Strategy I plants including dicot and non-graminaceous monocot species acquire Fe mainly by three processes including rhizospheric acidification (plasma membrane-localized H^+^-ATPase, AHA2; Santi and Schmidt, 2009), Fe^3+^ reduction (ferric chelate reductase, FRO2; Robinson et al., 1999), and Fe^2+^ transport (iron-regulated high-affinity transporter, IRT1; Varotto et al., 2002). Furthermore, Fe can go through long-distance transport from roots to shoots by several crucial genes such as *FRD3* (Green and Rogers, 2004), *NAS* (Haydon and Cobbett, 2007), and *YSL* (Waters et al., 2006). Strategy II plants including graminaceous species acquire Fe by root secretion of the mugineic acid (MA) family of phytosiderophores (PCs) to chelate Fe^3+^ (Jolley and Brown, 1989). Compared with strategy I plants, the strategy II plants exhibit better growth performance under alkaline stress, since the PCs-dependent Fe uptake is less susceptible to high pH conditions (Römheld and Marschner, 1986; Nozoye et al., 2011). However, the protons released by the strategy I plants are largely buffered by alkaline stress, and thus reducing the availability of soil Fe (Ohwaki and Sugahara, 1997). Importantly, exogenous abscisic acid (ABA) remarkably mitigates Fe deficiency-induced leaf chlorosis in plants by enhancing translocation of Fe from roots to shoots, indicating that high ABA levels may be beneficial to improve alkaline tolerance in plants by regulating Fe translocation (Lei et al., 2014).

Besides low availability of Fe, plants experiencing saline–alkaline stress resulting from high Na^+^ concentrations encounter problems such as osmotic imbalance and ion toxicity (Yang et al., 2007; Wang et al., 2012). Na^+^ toxicity is often associated with the systemic dysfunctions of uptake and distribution of K^+^ in plants (Shabala and Cuin, 2008; Anschütz et al., 2014). Numerous studies have indicated that saline-tolerant plants can effectively control intracellular K^+^ and Na^+^ balance, which is required for the stability of membrane potential and enzymatic activities (Anschütz et al., 2014; Zhang et al., 2016). The cytosol of saline-tolerant plants can maintain high K^+^ and low Na^+^ under salt stress. Recently, ABA has been shown to regulate the expression of tonoplast Na^+^/H^+^ antiporter genes, thereby modulating K^+^ and Na^+^ homeostasis (Fukuda et al., 2011).

Importantly, nitric oxide (NO) serves as a secondary messenger of ABA to enhance salt tolerance by increasing the K^+^/Na^+^ ratio (León et al., 2014). It has been indicated that plants seem to own the priming-like mechanisms that memorize the foregoing NO exposure events and activate defensive responses following harmful conditions (Tanou et al., 2009). Zhang et al. (2009) have reported that transgenic tobacco plants with high ABA levels are more tolerant to the controls under salt stress, which is closely associated with the ABA-induced NO accumulation. NO exposure markedly increases the tolerance of plants to Fe deficient conditions by efficient mobilization of cell wall Fe and activation of Fe deficiency-induced transcription factor 1 (FIT1) that regulates Fe uptake in plants (Graziano and Lamattina, 2007; Chen et al., 2010; Wang et al., 2017). Thus, plants with high level of ABA may possess more efficient systems to acquire Fe and detoxify Na^+^ for resisting the saline–alkaline stress.

Recently, growing attention has been attracted to beneficial soil bacteria present in plant rhizosphere. These rhizosphere-inhabiting microbes are collectively referred to as plant growth promoting rhizobacteria (PGPR) that have been widely employed in modern agriculture (Yuan et al., 2013; Mishra et al., 2014; Zebelo et al., 2016). A large number of studies have shown that PGPR strains can interact with plants, and control plant growth and pathogen invasion by synthesizing some growth regulators such as polyamines, hormones, and antibiotic substances (Dey et al., 2004; Scholz et al., 2011; Zhou et al., 2016b). So far, many works have been made to enhance the adaptation of plants to various abiotic stresses such as drought and salt stress by application of PGPR (Ait Barka et al., 2006; Sukweenadhi et al., 2015; Zhou et al., 2016b). However, lack of researches exists on the information about PGPR-induced saline–alkaline tolerance in plants.

*Chrysanthemum* is the most economically important medicinal and ornamental plants worldwide. Chuju, a cultivar of *Chrysanthemum morifolium*, has been widely exploited for drink and medicinal applications, and is ranked the first among the four famous *Chrysanthemum* plants in China (Xie et al., 2012). However, saline–alkaline soils often lead to plant growth inhibition and yield loss. Here, the main aim of this study tried to increase the tolerance of *Chrysanthemum* plants to saline–alkaline conditions by application of PGPR. The inoculation of *Chrysanthemum* plants with *Bacillus licheniformis* SA03 displayed better growth performance under saline–alkaline stress compared with non-inoculated plants. Moreover, we explored the underlying mechanisms at the physiological and molecular levels responsible for SA03-induced stress tolerance of plants.

## Materials and Methods

### Plant Materials, Growth Conditions, and Bacterial Inoculation

Seeds of *C. morifolium* cv. Chuju were surface sterilized in 0.1% (w/v) mercury dichloride (HgCl_2_), followed by rinsing at least three times with sterile water and placed on half-strength (1/2) MS medium containing 0.8% (w/v) agar and 1.5% (w/v) sucrose. The sterilized seeds were vernalized for 48 h at 4°C in darkness, and were then cultured in a growth chamber at 25°C with a photoperiod of 14 h light/10 h dark (light intensity of 200 μmol m^-2^ s^-1^). After 10 days (d) of germination, the seedlings were transferred into pots with soils (3:1:1, clay:vermiculite:perlite). Soils were sterilized by autoclaving at 120°C for 1 h before using it.

*Bacillus licheniformis* SA03 was isolated from the rhizospheric soils of *Chrysanthemum* plants grown under saline–alkaline conditions, and identified by 16S rDNA sequencing (GenBank No. KY828223). This bacteria strain was inoculated into MCF liquid medium (Freitas et al., 2015), and incubated in an orbital shaker (200 rpm) at 28°C for 18 h. Bacteria were collected by centrifugation at 8000 rpm at 4°C for 15 min and the centrifugal tubes were washed with 0.1 M phosphate-buffered saline (PBS, pH 7.2), and were then diluted to an OD570 nm absorbance of 0.7 in PBS buffer for microbial inoculation.

### Experiments with Pot-Grown Plants under Saline–Alkaline Stress

About 3-month-old *Chrysanthemum* plants were inoculated with 3 ml of PBS containing *B. licheniformis* SA03 or with 3 ml of PBS (as controls). After 10 days of co-culture, these plants were irrigated with sterile water containing 50 mM NaHCO_3_ and 50 mM Na_2_CO_3_ that allowed pH value of soils to reach approximate 8.2. After that, these plants were daily watered until plant tissues were harvested. Lastly, the harvested samples were used for various physiological and biochemical analyses.

### Assays of Metal Ion Contents

To measure metal ion content, about 500 mg of shoots and roots were firstly separated from *Chrysanthemum* plants, respectively. Then, the dried samples were ground and digested with 15 ml nitric acid (HNO_3_)/perhydrol (H_2_O_2_) (3:1, v/v) in a microwave system (MARS, CEM) at 160°C for 20 min. After centrifugation at 12,000 rpm for 10 min, the supernatant was used for determining metal ion contents by inductively coupled plasma-atomic emission spectroscopy (ICP-AES, Thermo Scientific, Waltham, MA, United States) as described recently by Lei et al. (2014).

### Determination of Photosynthetic Parameters

To examine total chlorophyll content in *Chrysanthemum* leaves, 500 mg of leaf samples was harvested and extracted with 5 ml of aqueous acetone (80%, v/v), and then centrifuged at 12,000 rpm for 15 min. Absorbance of the supernatant was recorded at wavelengths of 645 and 663 nm, respectively. The amounts of chlorophyll in leaves were calculated according to the formulae: 8.02 × A663 + 20.21 × A645 as reported previously by Porra (2002).

We further measured several chlorophyll fluorescence parameters including net photosynthetic rate (Pn), the effective quantum yield of PSII photochemistry (ΦPSII), and the ratio of the variable and the maximum chlorophyll fluorescence (*F*v/*F*m) according to the method described recently by Du et al. (2015).

### Measurement of Physiological Parameters

The content of two major types of reactive oxygen species (ROS) including $O_{2}^{•–}$ and H_2_O_2_ was measured according to the method described by Liu and Pang (2010). The values of MDA and EL were determined according to the method reported by Jiang and Zhang (2001). Moreover, the activities of antioxidant enzymes were assayed according to the methods reported by Mostofa et al. (2015).

Abscisic acid was firstly extracted and purified from shoots and roots, respectively. Then, the ABA content was determined by an indirect ELISA technique as described by Zhang et al. (2009). The NO content was measured according to the method described by Cvetkovska et al. (2014).

### RNA-Sequencing (RNA-Seq) Analysis

Total RNA was extracted from roots of the non-inoculated (NI) and inoculated (I) plants using Trizol reagent (Invitrogen, United States) following the manufacturer’s instructions. Residual DNA in total RNA was further digested by DNase (Invitrogen, United States). Then, the quality of integrity of RNA samples were analyzed using Agilent 2100 Bioanalyser (Agilent, United States). Total RNA from three independent plants in each group was used to construct two cDNA libraries in parallel using the Illumina TruSeq^TM^ RNA-seq library prep kit (Illumina, United States) according to the method described by Sun et al. (2016). The two cDNA libraries were sequenced using the Hiseq 2500 platform (Illumina, United States). The sequencing raw data were processed by removing the low-quality reads, and then submitted into the NCBI database (SRA^1^).

Gene direction and functions were annotated based on the Nr annotations. Gene ontology (GO) annotations with the default parameters were analyzed by the Blast2GO program, which were clustered into three groups including biological process, cellular component, and molecular function. Furthermore, the identification of differentially expressed genes (DEGs) between the NI and I libraries was conducted using a rigorous algorithm at false discovery rate (FDR)-adjusted *p*-value < 0.05. GO term^2^ was assigned to DEGs based on the above GO annotations. In addition, GO enrichment analysis was performed to search significantly enriched functional classification.

### Real-time Quantitative PCR (qRT-PCR)

Total RNA were extracted from roots using Trizol reagent (Invitrogen, United States), and genomic DNA contamination in RNA samples were digested by DNase (Promega, United States). Then, about 500 ng of total RNA was reversely transcribed into first-strand cDNA using a PrimeScript^®^ RT Reagent kit (TaKaRa, Japan) following the manufacturer’s instructions. qRT-PCR reactions were performed in a ABI 7500 real-time PCR machine. Each reaction contained 10 μl of 2 × SYBR Green Master Mix reagents, 1 μl of cDNA samples, and 0.5 μl of 10 μM primers in a final volume of 20 μl. The reaction conditions were as follows: 95°C for 30 s, followed by 40 cycles of 95°C for 15 s, 60°C for 30 s, and 72°C for 30 s. The *GAPDH* gene was used as an internal control to normalize target gene expression. Each experiment was conducted in three biological replicates. Each biological replicate was run with three independent cDNA samples. Gene specific primers for *IRT1, FRO2, FRD3, NHX1, NHX2, NHX5, AHA2, ZEP1, YSL1, YSL2, SAUR21, NAS1*, and *GAPDH* were listed in **Supplementary Table S1**.

### Ultrastructural Observation

Leaf samples were separated and cut into 0.5 cm × 0.5 cm pieces, and then fixed with 2.5% glutaraldehyde for 12 h. After at least three rinses with 0.1 M PBS, the samples were fixed with 1.0% osmium tetroxide (OsO4) for 2 h, followed by three rinses with PBS. Subsequently, the samples were dehydrated in an acetone dilution series from 30 to 100%, embedded in Spurr’s resin (Ted Pella, United States), and cut into thin sections (70–90 nm). Lastly, these sections were observed by transmission electron microscopy at 80 kV. At least five dependent samples and more than 16 individual chloroplasts were observed for each treatment.

### Statistical Analysis

Data were analyzed using SAS statistical software (SAS Institute, Cary, NC, United States), and were represented as the mean values ± SE. Significant differences were analyzed by one-way or two-way ANOVA followed by the Duncan’s multiple range test at *P* < 0.05.

## Results

### *B. licheniformis* SA03 Enhances Saline–Alkaline Tolerance in *Chrysanthemum* Plants

**FIGURE 1:**
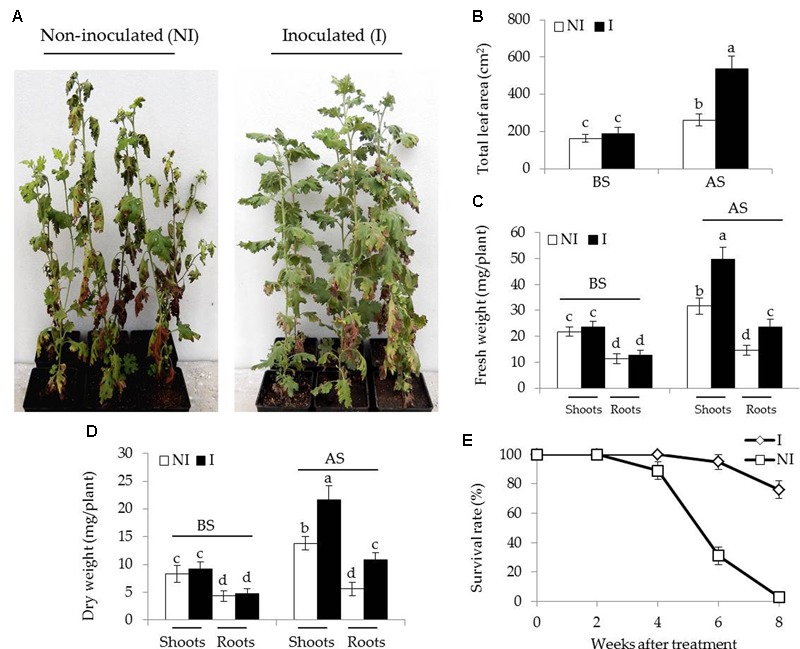
Effects of *B. licheniformis* SA03 on *Chrysanthemum* plants under saline–alkaline stress. After 10 days of bacterial inoculation, the non-inoculated (NI) and inoculated (I) were subjected to saline–alkaline treatment for 4 weeks, respectively. The treated plants were used to analyze **(A)** growth phenotype, **(B)** total leaf areas (LA), **(C)** shoot and root fresh weight, **(D)** shoot and root dry weight, and **(E)** survival rate. BS, before the stress treatment; AS, after the stress treatment. Data are expressed as the mean values of three replicates (±SE) with 10 plants each. Different letters indicate significant differences using two-way ANOVA followed by the Duncan’s multiple range test at *P* < 0.05.

To examine the effects of SA03-inoculation on the growth of *Chrysanthemum* plants under saline–alkaline stress, about 3-month-old plants were inoculated with this bacteria strain. After 10 days of co-culture, the non-inoculated and inoculated plants were subjected to saline–alkaline treatment for 4 weeks. There was no significant difference between the non-inoculated and inoculated plants before saline–alkaline treatment. However, the non-inoculated plants exhibited curly and yellowing leaves under saline–alkaline stress, while leaf chlorosis was hardly observed in the inoculated plants (**Figure 1A**). Several physiological parameters including total leaf area (LA), fresh and dry weight of plants were further examined. Soil inoculation greatly elevated about two-fold total LA in plants under the stress compared with the non-inoculated plants [**Figure 1B**; *F*(3,36) = 164.60, *P* < 0.05]. The inoculation with SA03 also led to 45 and 32% increase of shoot and root fresh weight, respectively [**Figure 1C**; *F*(7,72) = 296.99, *P* < 0.05]. Similarly, dry weight was pronouncedly increased in the inoculated plants compared with the non-inoculated plants [**Figure 1D**; *F*(7,72) = 146.08, *P* < 0.05]. Survival rates of plants were calculated until 8 weeks after saline–alkaline treatment. Soil inoculation resulted in a great increase of survival rates in the stress-treated plants. The non-inoculated plants grown in saline–alkaline soils for 8 weeks did not survive, while the survival rates of inoculated plants were about 76% (**Figure 1E**).

### SA03 Augments Photosynthesis and Fe Accumulation in Stress-Treated Plants

**FIGURE 2:**
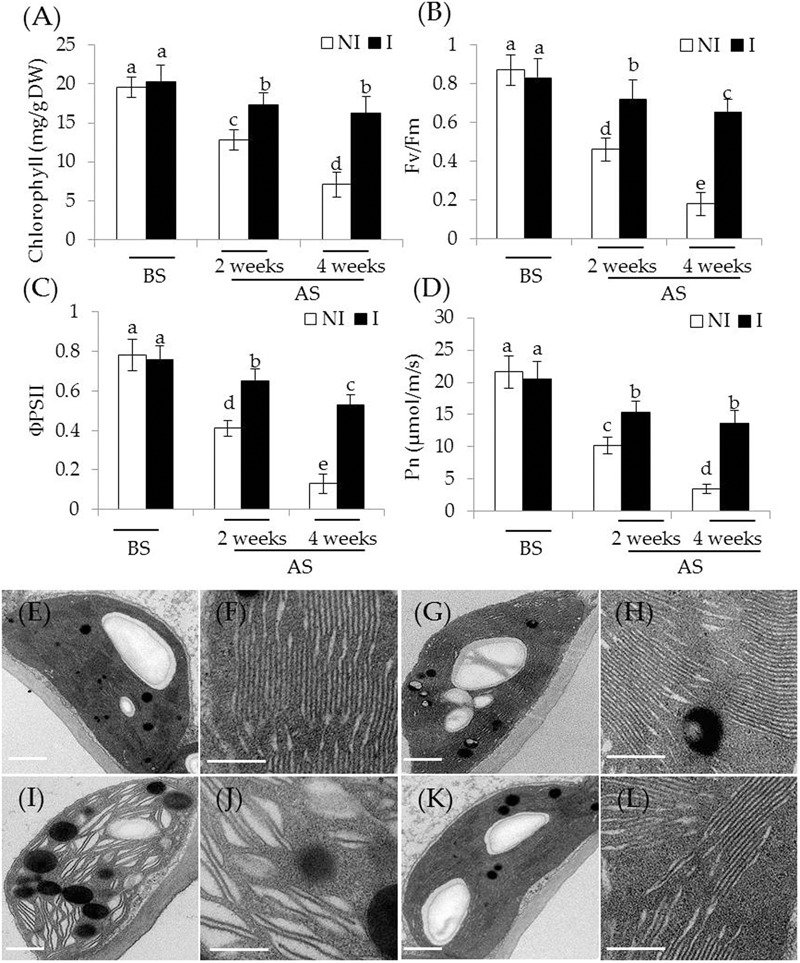
Effects of *B. licheniformis* SA03 on the photosynthesis of *Chrysanthemum* plants under saline–alkaline stress. After 10 days of bacterial inoculation, the non-inoculated (NI) and inoculated (I) were subjected to saline–alkaline treatment for 2 and 4 weeks, respectively. The treated plants were used to measure **(A)** total chlorophyll content; **(B)** *F*v/*F*m; **(C)** ΦPSII; and **(D)** Pn. In addition, transmission electron micrographs of chloroplast ultrastructure in mesophyll cells of plants. Chloroplast ultrastructure of the non-inoculated **(E)** and inoculated plants **(G)** before the stress treatment, and **(F,H)** indicated locally amplified view in **(E,G)**, respectively. Chloroplast ultrastructure of the non-inoculated **(I)** and inoculated plants **(K)** after the stress treatment, and **(J,L)** indicated locally amplified view in **(I,K)**, respectively. Scale bar = 1 μm. BS, before the stress treatment; AS, after the stress treatment. Data are expressed as the mean values of three replicates (±SE) with 10 plants each. Different letters indicate significant differences using two-way ANOVA followed by the Duncan’s multiple range test at *P* < 0.05.

To examine whether the inoculation with SA03 improved photosynthetic capacity in plants, several parameters associated with photosynthesis were examined. Under non-stress condition, there was indistinct difference between the non-inoculated and inoculated plants (**Figures 2A–D**). After 2 and 4 weeks of the stress treatment, total chlorophyll content was markedly decreased in the non-inoculated plants. However, the inoculated plants displayed higher chlorophyll levels under the stress compared with the non-inoculated plants [**Figure 2A**; *F*(5,54) = 84.31, *P* < 0.05]. In accordance with this, soil inoculation significantly increased photosynthetic efficiency in the stress-treated plants. The values of *F*v/*F*m, a pivotal index for the efficiency of PSII photochemistry, were also remarkably increased in the inoculated plants under the stress compared with the non-inoculated plants [**Figure 2B**; *F*(5,54) = 124.74, *P* < 0.05]. Similar results were observed for ΦPSII [**Figure 2C**; *F*(5,54) = 143.80, *P* < 0.05] and Pn [**Figure 2D**; *F*(5,54) = 118.43, *P* < 0.05]. After 4 weeks of saline–alkaline treatment, the non-inoculated plants displayed fully swollen chloroplasts and more rudimentary grana lamellae in plastids of mesophyll cells, but the number of normal grana stacking and grana lamellae was greater in the inoculated plants (**Figures 2E–L**).

The availability of Fe is exceedingly low in alkaline soils due to its immobilization (Römheld and Marschner, 1986). This often becomes a key limiting factor for plant growth and development. Thus, plants that are more tolerant to alkaline stress may equip with efficient systems to mine Fe from soils. To verify this hypothesis, shoot and root Fe concentrations were measured. Under non-stress condition, no striking difference in shoot Fe concentrations was observed between the non-inoculated and inoculated plants (**Table 1**). However, a marked decrease in shoot Fe concentrations was found in the non-inoculated plants after 2 and 4 weeks of the stress treatment [**Table 1**; *F*(5,54) = 57.42, *P* < 0.05]. Furthermore, shoot Fe concentrations of the inoculated plants were relatively higher than that of the non-inoculated plants. A similarly changing tendency of Fe concentrations was found in roots [**Table 1**; *F*(5,54) = 86.68, *P* < 0.05].

**Table 1:** Effects of *B. licheniformis* SA03 on shoot and root Fe concentrations in *Chrysanthemum* plants before (BS) or after (AS) saline–alkaline treatments.

Shoots (μg/g DW)											
	**BS**				**AS (2 weeks)**				**AS (4 weeks)**			
	**Mean**	***SE***	***n***	**^∗^**	**Mean**	***SE***	***n***	**^∗^**	**Mean**	***SE***	***n***	**^∗^**

NI	0.38	0.05	10	a	0.02	0.04	10	c	0.16	0.05	10	c
I	0.41	0.04	10	a	0.32	0.02	10	b	0.29	0.03	10	b
**Roots (μg/g DW)**					
NI	6.32	0.62	10	a	3.23	0.37	10	c	2.12	0.43	10	d
I	6.65	0.71	10	a	4.81	0.56	10	b	4.62	0.67	10	b

*Treatments and statistical analysis were as described in **Figure 2**.*

### SA03 Effectively Regulates Cellular K^+^ and Na^+^ Homeostasis in Plants

**FIGURE 3:**
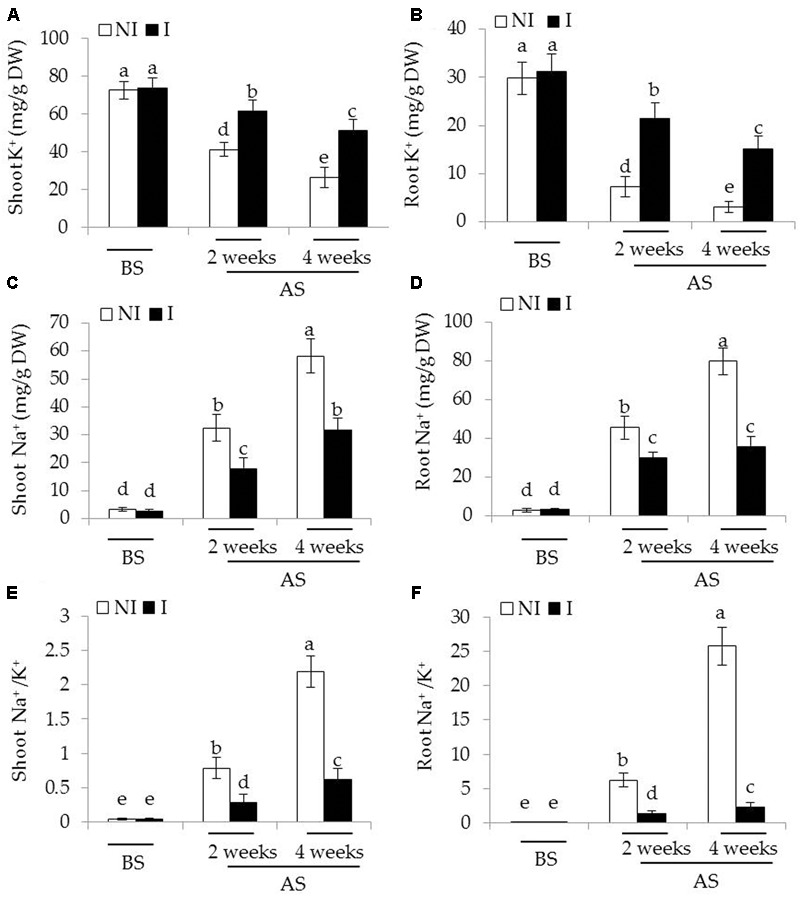
Effects of *B. licheniformis* SA03 on **(A,B)** K^+^ concentration, **(C,D)** Na^+^ concentration, and **(E,F)** Na^+^/K^+^ ratio of shoots and roots in *Chrysanthemum* plants before (BS) or after (AS) saline–alkaline treatments. Treatments and statistical analysis were as described in **Figure 2**.

Considering that plants experiencing saline–alkaline stress have to counteract excess toxic Na^+^ (Zhang et al., 2015), the effects of saline–alkaline stress on Na^+^ and K^+^ concentrations as well as Na^+^/K^+^ ratios in plants were investigated. Before saline–alkaline treatment, no significant difference was observed between the non-inoculated and inoculated plants (**Figure 3**). After 2 and 4 weeks of the stress treatment, shoot K^+^ concentrations of the inoculated plants were 33 and 45% higher than that of the non-inoculated plants [**Figure 3A**; *F*(5,54) = 122.69, *P* < 0.05], and root K^+^ concentrations were 67 and 75% higher [**Figure 3B**; *F*(5,54) = 171.30, *P* < 0.05], respectively. On the contrary, shoot Na^+^ concentrations of the inoculated plants were 35 and 42% lower than those of the non-inoculated plants [**Figure 3C**; *F*(5,54) = 290.86, *P* < 0.05], and root Na^+^ concentrations of the inoculated plants were 30 and 56% lower [**Figure 3D**; *F*(5,54) = 510.83, *P* < 0.05], respectively. These further led to higher ratio of Na^+^/K^+^ in shoots [**Figure 3E**; *F*(5,54) = 426.03, *P* < 0.05] and roots [**Figure 3F**; *F*(5,54) = 640.31, *P* < 0.05] the non-inoculated plants than that of the inoculated plants under saline–alkaline stress, respectively.

### SA03 Mitigates Oxidative Damage to Plants under Saline–Alkaline Stress

**FIGURE 4:**
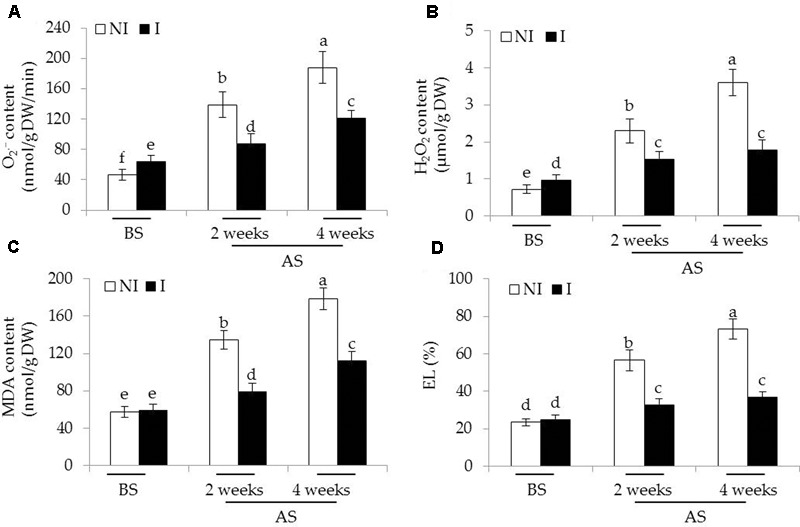
Effects of *B. licheniformis* SA03 on **(A)** $O_{2}^{•–}$, **(B)** H_2_O_2_ content, **(C)** MDA content, and **(D)** EL in *Chrysanthemum* plants before (BS) or after (AS) saline–alkaline treatments. Treatments and statistical analysis were as described in **Figure 2**.

To inspect whether the inoculated of plants with SA03 could confront oxidative stress imposed by saline–alkaline treatment, we measured the content of two major types of ROS including $O_{2}^{•–}$ and H_2_O_2_ in leaves of plants. Before saline–alkaline treatment, a slight increase of $O_{2}^{•–}$ [*F*(5,54) = 160.58, *P* < 0.05] and H_2_O_2_ [*F*(5,54) = 213.56, *P* < 0.05] levels was observed in the inoculated plants compared with the non-inoculated plants (**Figures 4A,B**). However, the values of MDA and EL, important indicators of oxidative damage, in both the non-inoculated and inoculated plants did not differ significantly (**Figures 4C,D**). After 2 and 4 weeks of the stress treatment, the content of $O_{2}^{•–}$ and H_2_O_2_ was markedly increased, especially in the 4-week treatment. By contrast, the inoculated plants displayed markedly lower content of $O_{2}^{•–}$ and H_2_O_2_. Consistent with this, saline–alkaline treatment considerably induced a great increase of MDA in plants. However, the leaves of inoculated plants had 36 and 32% lower MDA content than that of the non-inoculated plants under the stress, respectively [**Figure 4C**; *F*(5,54) = 311.48, *P* < 0.05]. Similarly, soil inoculation markedly decreased the EL levels in the leaves of inoculated plants under the stress compared with the non-inoculated plants [**Figure 4D**; *F*(5,54) = 253.81, *P* < 0.05].

**FIGURE 5:**
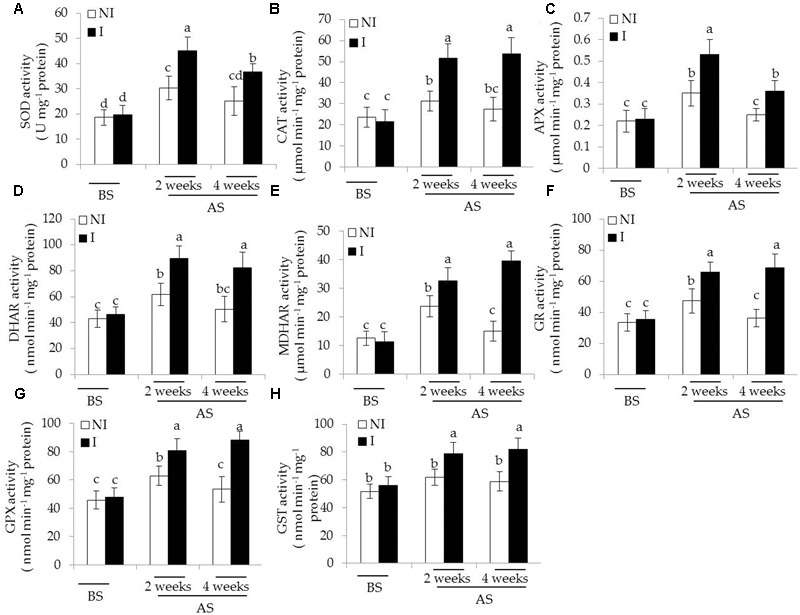
Effects of *B. licheniformis* SA03 on antioxidant enzymatic activities including **(A)** SOD, **(B)** CAT, **(C)** APX, **(D)** DHAR, **(E)** MDHAR, **(F)** GR, **(G)** GPX, and **(H)** GST in *Chrysanthemum* plants before (BS) or after (AS) saline–alkaline treatment. Treatments and statistical analysis were as described in **Figure 2**.

In plants, antioxidant enzymatic systems play essential roles in modulating dynamic homeostasis of cellular ROS, and thus detoxifying ROS effectively (Liu and Pang, 2010; Zhou et al., 2016b). Here, we investigated several antioxidant enzymatic activities in plants. The activities of antioxidant enzymes tested were no evident difference between the non-inoculated and inoculated plants before saline–alkaline treatment (**Figure 5**). After 2 and 4 weeks of the stress treatment, the activities of SOD, the first cellular defensive line converting $O_{2}^{•–}$ into H_2_O_2_, were significantly increased in the inoculated plants [**Figure 5A**; *F*(5,54) = 63.87, *P* < 0.05]. In contrast to the non-inoculated plants, the activities of CAT converting H_2_O_2_ to H_2_O and O_2_ were greatly higher in the inoculated plants [**Figure 5B**; *F*(5,54) = 48.78, *P* < 0.05]. The AsA-GSH cycle is an important metabolic pathway that eliminates H_2_O_2_ through multiple enzyme catalyzing reactions (Wei L. et al., 2015). The activities of APX [**Figure 5C**; *F*(5,54) = 42.93, *P* < 0.05], DHAR [**Figure 5D**; *F*(5,54) = 61.64, *P* < 0.05], MDHAR [**Figure 5E**; *F*(5,54) = 116.41, *P* < 0.05], and GR [**Figure 5F**; *F*(5,54) = 77.76, *P* < 0.05] were significantly enhanced in the inoculated plants under the stress compared with the non-inoculated plants. Similar results were also observed for the activities of GSH metabolizing enzymes including GPX [**Figure 5G**; *F*(5,54) = 79.77, *P* < 0.05] and GST [**Figure 5H**; *F*(5,54) = 26.36, *P* < 0.05].

### Transcriptomic Profiles of SA03-Inoculated Plants under Saline–Alkaline Stress

To clarify the mechanisms underlying SA03-induced saline–alkaline tolerance in plants, gene transcriptional profiles were analyzed by RNA-Seq. A total of 28,837,670 and 29,075,976 raw reads were generated in both the non-inoculated and inoculated plants by 454 sequencing, respectively (**Supplementary Table S2**), and the raw reads data were submitted into the NCBI SRA database (accession No. SRR5388903). After filtering out low quality reads, 23,021,019 (79.82%) and 22,541,540 (77.52%) clean reads were remained in both the NI and I library, respectively. Moreover, 22,003,842 clean reads (76.3%) in the NI library and 21,924,113 clean reads (75.4%) in the I library were uniquely mapped. We further compared analyses of gene expression between the non-inoculated and inoculated plants for screening DEGs with an FDR-adjusted *p*-value < 0.05 as the threshold (**Supplementary Table S3**).

**FIGURE 6:**
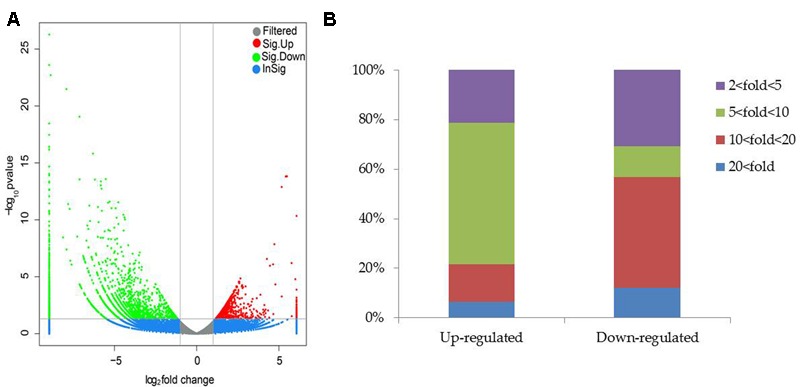
Distribution of differentially expressed genes between the non-inoculated (NI) and inoculated (I) libraries. **(A)** Scatter plot of gene expression difference. Green and red dots indicate up- and down-regulated genes under saline–alkaline stress, respectively. Blue dots indicate genes without significant differential expression, and gray dots indicate genes that were filtered out low quality reads. **(B)** Statistics of DEGs. Different color column indicated DEGs with diverse fold changes.

**FIGURE 7:**
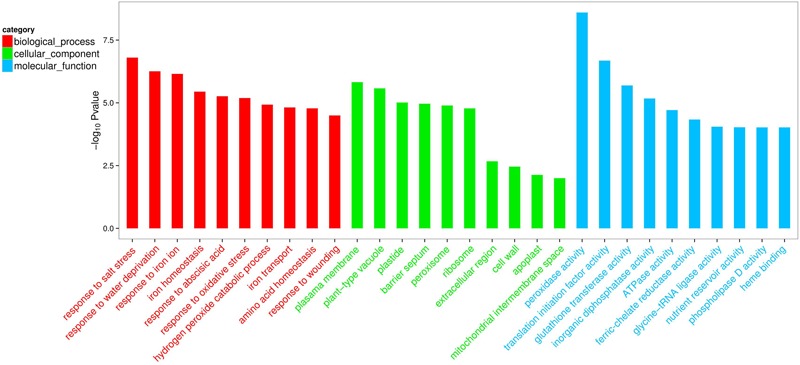
List of top 10 significant GO terms for up-regulated differentially expressed genes (DEGs) of SA03-inoculated plants based on GO classifications. GO terms were categorized into three groups: biological process, cellular component and molecular function. The *P*-value indicates the significance of the comparison between the non-inoculated and inoculated plants.

Compared with the non-inoculated plants, there were 693 up-regulated unigenes and 2456 down-regulated unigenes in the inoculated plants (**Figure 6**). Moreover, up- and down-regulated unigenes were aligned to the GO and COG database for classifying and predicting gene functions. GO term annotations for these up-regulated unigenes were classified to three major categories including molecular function, cellular component and biological process. Of these, assignments to biological process constituted the majority, followed by cellular component and molecular function (**Figure 7**). The most overrepresented categories in the up-regulated DEGs were mainly related to some important pathways such as ‘response to salt stress,’ ‘response to water deprivation,’ ‘response to iron ion,’ and ‘hydrogen peroxide catabolic process’ in biological processes; ‘plasma membrane,’ ‘plant-type vacuole,’ ‘plastide,’ and ‘peroxisome’ in cellular component; ‘peroxidase activity,’ ‘glutathione transferase activity,’ ATPase activity,’ and ‘ferric-chelate reductase activity’ in molecular function. Among these up-regulated DEGs, some genes involved in Fe acquisition, Na^+^ transport and antioxidant systems were observably activated in the inoculated plants (**Supplementary Table S4**). Thus, the enhanced stress tolerance of plants by SA03 was closely associated with multiple signaling pathways involving Fe uptake and stress adaption. Moreover, qRT-PCR was used to confirm gene expression profiles that were found in the DEGs (eight unigenes: *IRT1, FRD3, NHX1, AHA2, ZEP1, YSL1, SAUR21*, and *NAS1*). The changing patterns of gene expression were in accordance with that detected by RNA-Seq (**Supplementary Figure S1**), indicating a high reliability of RNA-Seq data.

### Involvement of ABA and NO in SA03-Induced Stress Tolerance in Plants

**FIGURE 8:**
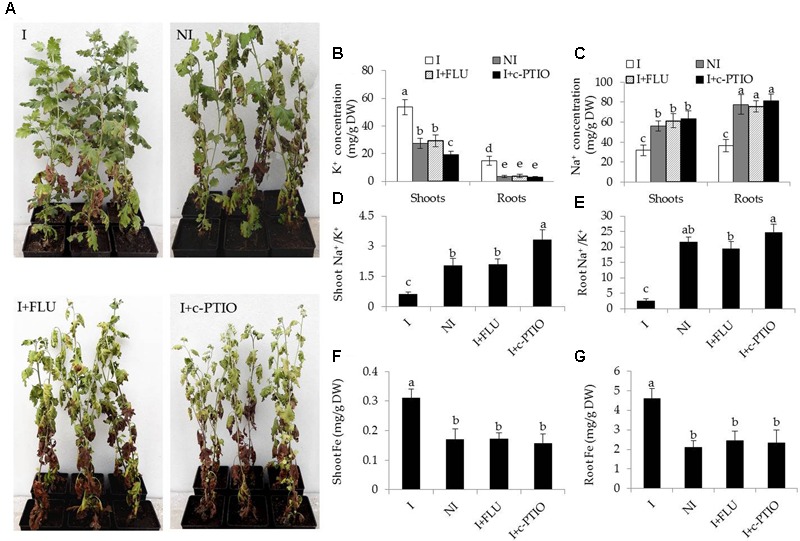
Treatment with FLU or c-PTIO abrogated the effects of *B. licheniformis* SA03 on saline–alkaline tolerance of *Chrysanthemum* plants. After 10 days of bacterial inoculation, plants were subjected to saline–alkaline stress for 4 weeks with or without FLU or c-PTIO treatments. These plants were used to analyze **(A)** growth phenotypes, shoot and root **(B)** K^+^ or **(C)** Na^+^ concentration, shoot **(D)** and root **(E)** Na^+^/K^+^ ratio, shoot **(F)** and root **(G)** Fe concentrations. I, inoculated plants; NI, non-inoculated plants; BS, before the stress treatment; AS, after the stress treatment. Data are expressed as the mean values of three replicates (±SE) with 10 plants each. Different letters indicate significant differences using one-way ANOVA followed by the Duncan’s multiple range test at *P* < 0.05.

Some hormone signaling pathways in host plants are tightly regulated by some PGPR strains, and thus affecting various physiological processes (Poupin et al., 2016; Zhou et al., 2016a). ABA plays a cardinal role in abiotic stress responses in plants (Zhang et al., 2006). In this study, transcriptomic analyses indicated that the inoculation with SA03 might affect cellular ABA levels in plants, and further regulate stress-related signaling pathways. To test the hypothesis, the ABA content in plants was measured. The roots of inoculated plants exhibited slightly higher ABA content than that of non-inoculated plants before saline–alkaline treatment, whereas no significant difference was observed in shoots (**Table 2**). After 2 and 4 weeks of saline–alkaline treatment, the ABA content was significantly higher in shoots [*F*(5,54) = 369.14, *P* < 0.05] and roots [*F*(5,54) = 123.34, *P* < 0.05] of the inoculated plants than that of the non-inoculated plants (**Table 2**). Furthermore, we explored the effects of fluridone (FLU), an inhibitor of ABA biosynthesis, on the inoculated plants under saline–alkaline stress. It was observed that treatment with 10 μM FLU abolished the SA03-induced stress tolerance of plants (**Figure 8A**). Many studies have indicated that NO serves as an important signal molecule to regulate abiotic stress responses, Fe uptake and remobilization in plants (Graziano and Lamattina, 2007; Tanou et al., 2009; Chen et al., 2010; Wang et al., 2017). For this reason, we examined if NO was involved in the SA03-mediated stress responses in plants. Intriguingly, the inoculation with SA03 could not increase the tolerance of plants to saline–alkaline stress after treatment with 150 μM c-PTIO, a scavenger of NO. That was similar to the phenotypes observed for the FLU-treated plants (**Figure 8A**).

**Table 2:** Effects of *B. licheniformis* SA03 on shoot and root ABA content in *Chrysanthemum* plants before (BS) or after (AS) saline–alkaline treatments.

Shoots (ng/g DW)											
	**BS**				**AS (2 weeks)**				**AS (4 weeks)**			
	**Mean**	***SE***	***n***	**^∗^**	**Mean**	***SE***	***n***	**^∗^**	**Mean**	***SE***	***n***	**^∗^**

NI	5.72	1.16	10	e	21.3	1.87	10	d	30.3	3.17	10	c
I	6.26	1.35	10	e	39.2	3.91	10	b	48.9	4.52	10	a
**Roots (ng/g DW)**					
NI	3.21	0.87	10	e	12.36	2.15	10	c	18.32	2.37	10	b
I	6.22	2.35	10	d	25.61	3.64	10	a	26.8	4.52	10	a

**FIGURE 9:**
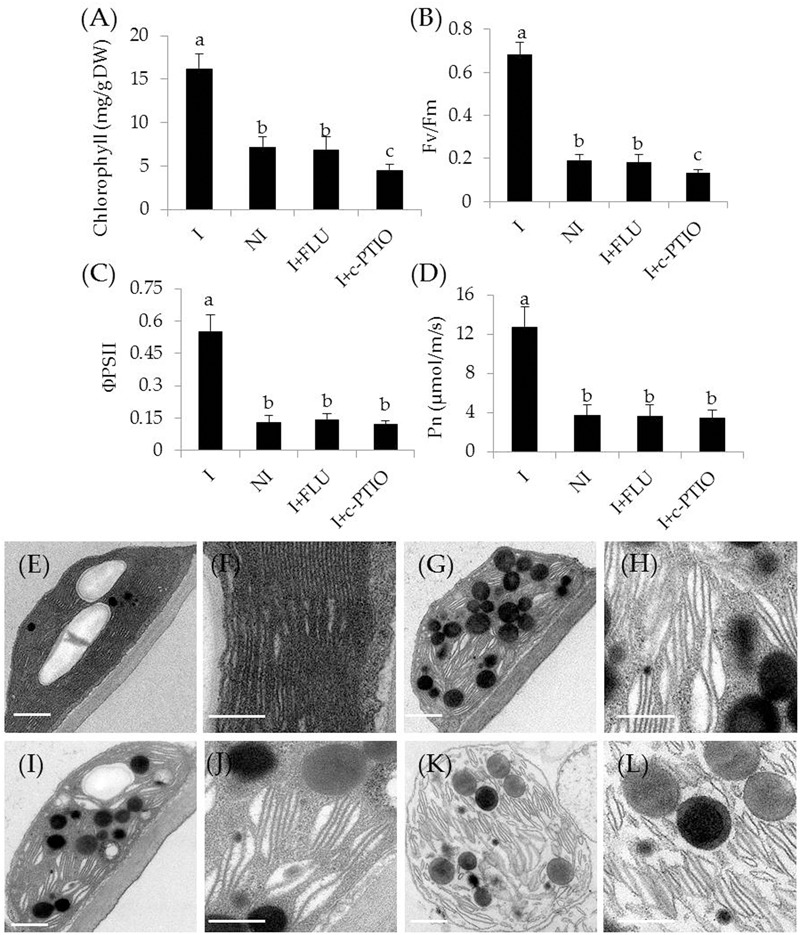
Treatment with FLU or c-PTIO affected **(A)** total chlorophyll content, **(B)** *F*v/*F*m, **(C)** ΦPSII, and **(D)** Pn. In addition, chloroplast ultrastructure of the non-inoculated and inoculated plants: **(E)** the inoculated plants, **(G)** the non-inoculated plants, and **(I)** FLU- or **(K)** c-PTIO-treated inoculated plants. **(F,H,J,L)** Indicated locally amplified views in **(E,G,I,K)**, respectively. Scale bar = 1 μm. Treatments and statistical analysis were as described in **Figure 8**.

In addition, some physiological parameters were determined in the stress-treated plants with FLU or c-PTIO treatment. After 4 weeks of FLU exposure, shoot and root Na^+^ concentrations [**Figure 8B**; *F*(7,72) = 333.37, *P* < 0.05] were significantly increased in the inoculated plants under saline–alkaline stress, but shoot and root K^+^ concentrations [**Figure 8C**; *F*(7,72) = 94.66, *P* < 0.05] was evidently decreased in the inoculated plants under the stress, thereby leading to higher shoot [**Figure 8D**; *F*(3,36) = 89.93, *P* < 0.05] and root [**Figure 8E**; *F*(3,36) = 231.41, *P* < 0.05] Na^+^/K^+^ ratios. However, there was no significant difference in these parameters between the non-inoculated and FLU-treated inoculated plants. Furthermore, shoot Fe concentrations of non-inoculated plants did not differ significantly from the FLU-treated inoculated plants (**Figure 8F**). Similarly, root Fe concentrations were no significant difference between the non-inoculated and FLU-treated inoculated plants (**Figure 8G**). Moreover, the Na^+^ and K^+^ concentrations, Na^+^/K^+^ ratios as well as Fe concentrations were also determined in c-PTIO-treated plants. The changing tendency of physiological parameters in the c-PTIO-treated inoculated plants displayed the similarities with the results observed for the FLU-treated inoculated plants, whereas these negative effects was notably aggravated by c-PTIO treatment. Concomitantly, either FLU or c-PTIO treatment significantly reduced total chlorophyll content [**Figure 9A**; *F*(3,36) = 147.13, *P* < 0.05] and photosynthetic parameters including *F*v/*F*m [**Figure 9B**; *F*(3,36) = 422.56, *P* < 0.05], ΦPSII [**Figure 9C**; *F*(3,36) = 166.62, *P* < 0.05] and Pn [**Figure 9D**; *F*(3,36) = 107.68, *P* < 0.05] in the inoculated plants under the stress compared with the untreated inoculated plants. As the photosynthetic apparatus, fully swollen chloroplasts occurred in the leaves of non-inoculated plants under the stress, whereas chloroplast ultrastructure of the inoculated leaves was not severely damaged by saline–alkaline stress (**Figures 9E–L**). When plants were treated with FLU or c-PTIO, the number of grana stacking was evidently decreased in the inoculated plants under the stress. However, the damages were even further increased in the c-PTIO-treated inoculated plants. These results implied the roles of ABA and NO in the SA03-induced stress tolerance of plants.

### NO Acts Downstream of ABA to Regulate Plant’s Adaptive Responses

In this study, both ABA and NO seemed to participate in SA03-induced saline–alkaline tolerance of plants. This raised the question how ABA interacted with NO to regulate the adaptive responses of SA03-inoculated plants to saline–alkaline stress. Since ABA has been shown to regulate the NO accumulation in plants under salt stress (Zhang et al., 2009), we wondered if SA03-induced a great increase of ABA resulted in promoting NO biosynthesis. Indeed, we found that cellular NO levels were markedly higher in the inoculated roots compared with the non-inoculated plants under non-stress condition. Moreover, saline–alkaline treatment strikingly enhanced the NO biosynthesis in plants, whereas the inoculated plants accumulated more NO than the non-inoculated plants. Intriguingly, FLU exposure did not remarkably affect endogenous NO content in the inoculated plants under non-stress condition, whereas a marked decrease of NO levels was observed in the FLU-treated inoculated plants under the stress compared with the untreated inoculated plants (**Supplementary Figure S2**).

**FIGURE 10:**
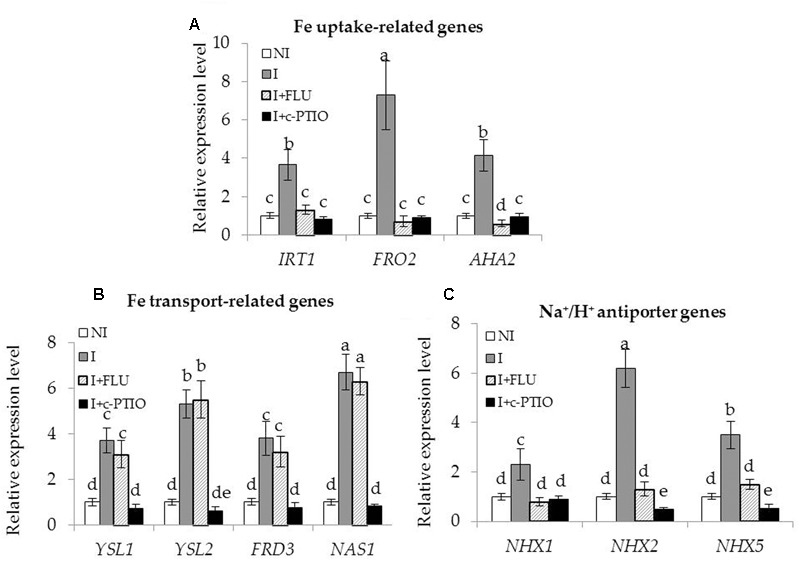
Treatment with FLU or c-PTIO affected the expression of Fe acquisition and Na^+^/H^+^ antiporter genes in SA03-inoculated *Chrysanthemum* plants. After 10 days of SA03-inoculation, plants were subjected to saline–alkaline stress for 4 weeks with FLU or c-PTIO treatment. These plants were used to examine the transcription of **(A)** Fe uptake-related (*IRT1, FRO2*, and *AHA2*), **(B)** Fe transport-related (*YSL1, YSL2, FRD3,* and *NAS1*), and **(C)** Na^+^/H^+^ antiporter (*NHX1, NHX2,* and *NHX5*) genes by qRT-PCR. Data are expressed as the mean values of three replicates (±SE). Different letters indicate significant differences using two-way ANOVA followed by the Duncan’s multiple range test at *P* < 0.05.

Enhanced Fe acquisition and reduced Na^+^ toxicity are required for improving the tolerance of plants to saline–alkaline stress (Li et al., 2016). In this study, the SA03-inoculated plants exhibited higher Fe accumulation and lower Na^+^ levels under saline–alkaline stress. qRT-PCR analyses showed that the transcription levels of Fe acquisition-related genes and Na^+^/H^+^ antiporter genes were significantly up-regulated in the inoculated plants compared with the non-inoculated plants. To clarify how microbial induction of ABA and NO regulated these adaptive responses, the transcription of genes associated with Fe acquisition and Na^+^/H^+^ antiporters were investigated in plants treated with FLU or c-PTIO. The expression levels of Fe uptake-related (*IRT1, FRO2*, and *AHA2*) [**Figure 10**; *F*(11,24) = 202.47, *P* < 0.05], Fe transport-related (*YSL1, YSL2, FRD3* and *NAS1*) [**Figure 10**; *F*(15,32) = 71.20, *P* < 0.05], and Na^+^/H^+^ antiporter (*NHX1, NHX2* and *NHX5*) [**Figure 10**; *F*(11,24) = 251.41, *P* < 0.05] genes were remarkably increased in the inoculated plants under the stress compared with the non-inoculated plants. However, their transcription levels were markedly down-regulated in the inoculated plants after FLU treatment. Upon exposure to c-PTIO, the transcription of Fe acquisition-related and Na^+^/H^+^ antiporter genes was greatly repressed in the inoculated plants, but no significant difference was observed for the transcription of Fe transport-related genes. Moreover, either FLU or c-PTIO treatment reduced the expression of Na^+^/H^+^ antiporter genes in the inoculated plants under the stress.

## Discussion

Along long-term evolution, plants have developed some flexible mechanisms to adapt to adverse environments (Marschner and Römheld, 1994; Chen et al., 2010; Li et al., 2016). It is one of important strategies to attract colonization of diverse beneficial microbes in the rhizosphere of host plants (Lebeis et al., 2015). It is widely recognized that the complex mutualistic interactions can assist plants to cope with unfavorable conditions (Ait Barka et al., 2006; Mishra et al., 2014; Sukweenadhi et al., 2015). Recently, numerous studies have indicated that PGPR confers increased tolerance of plants to various abiotic stresses including drought, salinity, and nutrient deficiency (Dey et al., 2004; Scholz et al., 2011; Zhou et al., 2016a,b). However, whether PGPR could induce saline–alkaline tolerance in plants and the underlying mechanisms remain elusive. We reported here for the first time that *Chrysanthemum* plants inoculated with *B. licheniformis* SA03 were greater resistant to saline–alkaline conditions, as evidenced by lower biomass loss and higher survival rates. Moreover, transcriptomic, biochemical, and pharmacological analyses were combined to unravel the mechanisms behind SA03 activated the adaptive responses of plants to saline–alkaline conditions. Our results revealed that the SA03-induced ABA accumulation was required for the tolerance of plants to saline–alkaline stress, indicating that the increased ABA level was a primarily acting mode of SA03 to regulate saline–alkaline stress response in plants.

Plant growth and productivity is tightly associated with adverse conditions, which cause a marked decrease in plant photosynthesis, thereby affecting its productivity (Li et al., 2016; Zhou et al., 2016b). In this study, in addition to greater biomass, the SA03-inoculated plants displayed higher chlorophyll content under saline–alkaline stress, which possibly led to stronger photosynthetic capacity. This positive effect may improve the growth performance of inoculated plants under saline–alkaline stress. A great photosynthetic efficiency in plants indicates that the photosynthetic apparatus is not considerably damaged by saline–alkaline treatment (Gong et al., 2014). It has recently been shown that the structural and functional integrity of chloroplasts are seriously destroyed by saline–alkaline stress (Li et al., 2016). In this study, the stress-treated plants displayed leaf chlorosis, indicating a serious disturbance of normal chloroplast development. Saline–alkaline stress has recently been shown to reduce the contents of chlorophyll and affect normal chloroplast development (Gong et al., 2014). Here, the inoculation with SA03 conspicuously lessened oxidative damages to chloroplast ultrastructure caused by saline–alkaline stress to a large extent, thereby leading to high photosynthetic efficiency. Furthermore, the non-inoculated plants exhibited the increased values of ROS and MDA under saline–alkaline stress, whereas their values were markedly lower in the inoculated plants. Additionally, the EL levels were relatively lower in the inoculated plants than the non-inoculated plants. Consistently, the inoculated plants owned higher ROS-detoxifying enzymatic activities than the non-inoculated plants. Ample evidence has indicated that the enhanced activities of antioxidant enzymes play important roles in modulating ROS levels in plants under adverse stresses (Gong et al., 2014; Wei L. et al., 2015; Zhou et al., 2016b). Therefore, these findings indicated that the inoculation with SA03 can alleviate oxidative damage imposed by saline–alkaline stress.

In fact, plants have to cope with two inevitable challenges including low availability of Fe and Na^+^ toxicity under saline–alkaline stress (Li et al., 2016). Fe often forms exceedingly insoluble hydroxides and oxides under alkaline conditions, thereby reducing its availability for plants (Romera and Alcántara, 2004). In this study, the mixtures of NaHCO_3_ and Na_2_CO_3_ were added into soils to mimic saline–alkaline conditions with high levels of Na^+^ and soil pH values. Overproduction of cellular ROS can be triggered by either Fe deficiency or high concentrations of Na^+^, which causes peroxidation of membrane lipid and proteins, and even cell death (Gong et al., 2014). Here, the SA03-inoculated plants experienced less ROS-mediated oxidative injury under saline–alkaline stress compared with the non-inoculated plants. This allowed us to conclude that SA03 conferred more efficient systems of plants to regulate the accumulation of Fe and Na^+^. To prove these assumptions, the Fe concentrations in both the non-inoculated and inoculated plants were firstly examined under saline–alkaline stress. A significant decrease of Fe concentrations was observed in shoots of the non-inoculated plants grown under the stress. However, the Fe concentrations remained relatively high in shoots of the inoculated plants. The results demonstrated that the SA03-inoculated plants equipped with an efficient system of Fe acquisition, thereby enhancing the adaptation of plants to Fe deficiency induced by saline–alkaline stress.

Besides low Fe bioavailability, high Na^+^ concentrations seriously disrupt plant growth in saline–alkaline soils, and thus exposing plants to saline stress (Wang et al., 2012). Saline-tolerant plants can effectively maintain a high K^+^ concentration and a concurrent low Na^+^ in their shoots, indicating that the Na^+^/K^+^ ratio is an index of salt tolerance in plants (Cui et al., 2016). Transgenic plants with high tolerance to salt stress display lower Na^+^/K^+^ ratio compared with wild-type plants (Cui et al., 2016; Zhang et al., 2016). Takahashi et al. (2007) have reported that the saline-tolerant reed plants have lower shoot Na^+^/K^+^ ratio than plants that are the most sensitive to saline stress. We observed here that shoots and roots of SA03-inoculated plants displayed relatively lower Na^+^/K^+^ ratio than non-inoculated plants under saline–alkaline stress, indicating that the enhanced saline–alkaline tolerance by SA03 was partially attributable to minimize Na accumulation. Exposure to high concentrations of Na^+^ has been shown to severely damage various enzymatic activities in plants (Gong et al., 2014). It is well documented that the increased ABA levels remarkably increases root net Na^+^ efflux and H^+^ influx, and decreases net K^+^ efflux in transgenic maize by activation of Na^+^/H^+^ antiporters and K^+^ channels (Zhang et al., 2016). Hence, the induced expression of Na^+^/H^+^ antiporter genes is a crucial strategy for plants to tolerate salt stress.

To elucidate the mechanisms underlying the SA03-induced stress tolerance of plants, comparative transcriptomic analyses were used to identify DEGs associated with the adaptive responses of plants to the stress. Here, the inoculation with SA03 activated several major biological processes associated with salt and drought stress, Fe acquisition, wounding, and response to ABA under saline–alkaline stress. It is well known that ABA plays a cardinal role in the regulation of abiotic stress responses and tolerance in plants. The increased ABA levels can activate a wide array of many stress-responsive genes in plants, which contributes to abiotic stress tolerance. In addition, ABA has been demonstrated to regulate Fe deficiency responses in *Arabidopsis* plants (Lei et al., 2014). Intriguingly, ABA can also enhance the activity of plasma membrane H^+^-ATPase to release protons along root tips (Xu et al., 2013). In this study, transcriptomic analyses showed that the ATPase activity was significantly increased in the SA03-inoculated plants under saline–alkaline stress. Recent studies have indicated that plasma membrane H^+^-ATPase plays a vital role in the adaptation of plant roots to alkaline conditions by modulating proton secretion (Fuglsang et al., 2007; Yang et al., 2010), indicating that the ABA-induced H^+^-ATPase activity can increase the release of protons into plant rhizosphere for counteracting adverse impacts imposed by alkaline pH conditions. More recently, some PGPR strains markedly induce ABA accumulation in host plants under abiotic stress (Salomon et al., 2014; Cohen et al., 2015; Park et al., 2017). Salomon et al. (2014) have reported that *B. licheniformis* Rt4M10 reduces water losses in drought-treated grapevine plants by induction of ABA synthesis. *Azospirillum brasilense* ameliorates drought stress in *Arabidopsis thaliana* mainly through enhancement of ABA levels (Cohen et al., 2015). Moreover, the inoculation with *Bacillus aryabhattai* SRB02 induces a great increase of ABA level and further activates ABA-mediated stomatal closure in soybean, which contributes to better heat stress tolerance (Park et al., 2017). These results have indicated that application of PGPR can enhance the tolerance of host plants to various abiotic stresses by activating ABA-mediated signaling pathways. Thus, the increased tolerance of SA03-inoculated plants against saline–alkaline stress may result from alteration of ABA levels.

To verify this hypothesis, the changing patterns of ABA content in plants were examined. As expectedly, a marked increase in ABA levels was observed in leaves and roots of non-inoculated plants as a consequence of saline–alkaline treatment. Also, the inoculated plants exhibited a great rise in ABA levels, displaying a similar pattern for the non-inoculated plants. However, more ABA accumulation was observed in the inoculated plants than the non-inoculated plants. Wei L.X. et al. (2015) have reported that ABA treatment can improve the tolerance of rice plants to alkaline stress. Hence, the SA03-induced ABA accumulation may be responsible for enhancing the adaptation of plants to saline–alkaline stress. We further examined if the inhibited ABA biosynthesis affected the SA03-induced stress responses in plants. When plants were treated with FLU, the inoculated plants exhibited similar phenotypes with the non-inoculated plants under saline–alkaline stress. Consistently, several physiological parameters such as photosynthesis and the accumulation of Na^+^, K^+^ and Fe were markedly altered in the inoculated plants under the stress, which indistinctly differed from the non-inoculated plants. Similar results are recently reported by Wei L.X. et al. (2015) in which FLU exposure markedly increases the degree of cell membrane injury and reduces relative water content under alkaline stress, indicating that ABA plays a vital role in mediating the adaptive responses of plants to alkaline stress. Additionally, qRT-PCR analyses revealed that FLU exposure pronouncedly down-regulated the transcription of some Fe acquisition- and Na^+^ transport-related genes, which was in accordance with the physiological parameters observed.

Interestingly, the inoculation with SA03 notably promoted the NO accumulation in the stress-treated plants accompanied by the increased cellular ABA levels. However, FLU exposure notably repressed the NO biosynthesis under saline–alkaline stress. It has previously been indicated that NO plays crucial roles in abiotic stress response and tolerance in plants, which is combined with ABA and other hormones (León et al., 2014). Moreover, NO acts as downstream signals of ABA to regulate salt tolerance in plants by activating antioxidant enzymatic activities and Na^+^/H^+^ antiporters (Zhang et al., 2009). Recently, NO has also been found to regulate Fe deficiency responses by remobilizing cell wall Fe and provoking the FIT1-mediated signaling pathways (Graziano and Lamattina, 2007; Chen et al., 2010; Wang et al., 2017). Thus, NO may function as a secondary messenger of ABA to activate diverse adaptive mechanisms that alleviate adverse effects caused by saline–alkaline stress. In this study, the inoculated plants treated with c-PTIO shared the resemblance in phenotypic traits and alteration of physiological parameters with the FLU-treated inoculated plants under the stress. Intriguingly, c-PTIO exposure did not markedly down-regulate the expression of Fe transport-related genes in the inoculated plants compared with the FLU-treated inoculated plants, although no significant difference in shoot Fe concentrations was observed between the c-PTIO- and FLU-treated plants under saline–alkaline stress. Previous studies have indicated that NO not only regulates FIT1-mediated *IRT1* and *FRO2*, but also can chelate Fe from cell wall (Chen et al., 2010). It is well known that about 75% of Fe is deposited in root cell walls, which are severed as largest reservoir for apoplastic Fe (Bienfait et al., 1985). Fe deficiency can trigger rapid accumulation of NO to invoke Fe uptake and reutilize cell wall Fe (Graziano and Lamattina, 2007). These indicated that inhibition of NO biosynthesis blocked plant uptake of Fe from rhizosphere soils and remobilization of apoplastic Fe from roots to shoots under Fe deficient conditions. This may explain the reason that the FLU-treated inoculated plants had high-level expressions of Fe transport-related genes under saline–alkaline stress, which could not lead to the increase in shoot Fe concentrations. Therefore, the SA03-induced stress tolerance of plants mainly attributed to the ABA-mediated NO signaling pathways.

## Conclusion

The inoculation of *Chrysanthemum* plants with *B. licheniformis* SA03 ameliorated the detrimental impacts caused by saline–alkaline stress by enhancement of the ABA levels. Soil inoculation sufficiently activated a series of adaptive mechanisms such as increased antioxidant enzymatic activities, enhanced Fe acquisition, and decreased Na^+^ accumulation in host plants, which were in correlation with the actions of NO. The findings confirmed the roles of SA03 in assisting host plants to saline–alkaline stress and its use as a potential strategy in sustainable agriculture.

## Author Contributions

JW and CZ conceived and designed the experiment; CZ, FL, and LZ performed the experiment; CZ, XX, YX, and ZM analyzed the data; CZ wrote the paper.

## Conflict of Interest Statement

The authors declare that the research was conducted in the absence of any commercial or financial relationships that could be construed as a potential conflict of interest.

## Footnotes

1 http://www.ncbi.nlm.nih.gov/Traces/sra

2 http://www.geneontology.org

## Supplementary Material

FIGURE S1 qRT-PCR analyses of some selective genes including IRT1, FRD3, NHX1, AHA2, ZEP1, YSL1, SAUR21, and NAS1 among the DEGs. Data are expressed as the mean values of three replicates (±SE). Different letters indicate significant differences using two-way ANOVA followed by the Duncan’s multiple range test at P < 0.05.

FIGURE S2 FLU treatment significantly decreased endogenous NO content in Chrysanthemum plants. After 10 days of bacterial inoculation, plants were subjected to saline–alkaline stress for the indicated times with or without exposure to FLU. These plants were used to analyze shoot (A) and root (B) NO content. I, inoculated plants; NI, non-inoculated plants. Data are the mean values of three replicates (±SE) with 10 plants each. Different letters indicate significant differences using two-way ANOVA followed by the Duncan’s multiple range test at P < 0.05.

TABLE S1 Primers used in this study.

TABLE S2 Statistics of RNA-sequencing and mapping.

TABLE S3 Up-regulated and down-regulated genes (more than two-fold) differentially expressed in the SA03-inoculated plants under saline-alkaline stress.

TABLE S4 Up-regulated differentially expressed genes (DEGs) including Fe acquisition-, Na^+^ transport-, and antioxidant-related genes in the SA03-inoculated plants under saline–alkaline stress.
