# Supplementary figures and images for: Bacillus licheniformis SA03 Confers Increased Saline–Alkaline Tolerance in Chrysanthemum Plants by Induction of Abscisic Acid Accumulation
Source: Front Plant Sci. 2017 Jun 29;8:1143. doi: 10.3389/fpls.2017.01143 (PMC5489591; doi:10.3389/fpls.2017.01143)

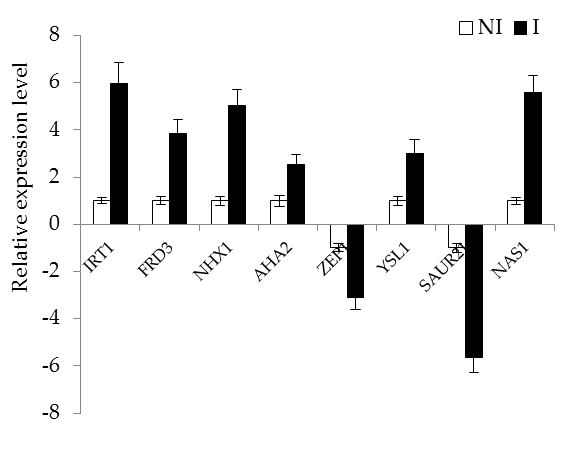

Supplement: FIGURE S1 — qRT-PCR analyses of some selective genes including IRT1, FRD3, NHX1, AHA2, ZEP1, YSL1, SAUR21, and NAS1 among the DEGs. Data are expressed as the mean values of three replicates (±SE). Different letters indicate significant differences using two-way ANOVA followed by the Duncan’s multiple range test at P < 0.05. [file Image_1.jpg]

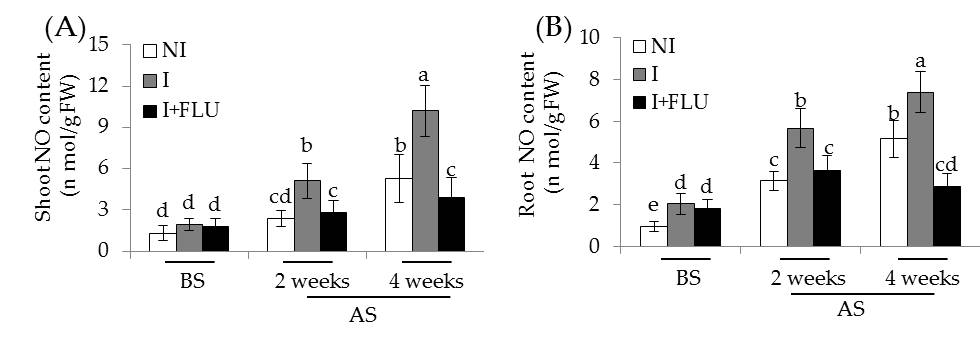

Supplement: FIGURE S2 — FLU treatment significantly decreased endogenous NO content in Chrysanthemum plants. After 10 days of bacterial inoculation, plants were subjected to saline–alkaline stress for the indicated times with or without exposure to FLU. These plants were used to analyze shoot (A) and root (B) NO content. I, inoculated plants; NI, non-inoculated plants. Data are the mean values of three replicates (±SE) with 10 plants each. Different letters indicate significant differences using two-way ANOVA followed by the Duncan’s multiple range test at P < 0.05. [file Image_2.jpg]
